# Using Rapid Invisible Frequency Tagging (RIFT) to Probe the Neural Interaction Between Representations of Speech Planning and Comprehension

**DOI:** 10.1162/nol_a_00171

**Published:** 2025-07-15

**Authors:** Cecília Hustá, Antje Meyer, Linda Drijvers

**Affiliations:** Max Planck Institute for Psycholinguistics, Nijmegen, The Netherlands; Donders Institute for Brain, Cognition and Behaviour, Radboud University, Nijmegen, The Netherlands

**Keywords:** attention, comprehension, electroencephalography (EEG), interaction, rapid invisible frequency tagging (RIFT), speech planning

## Abstract

Interlocutors often use the semantics of comprehended speech to inform the semantics of planned speech. Do representations of the comprehension and planning stimuli interact? In this EEG study, we used rapid invisible frequency tagging (RIFT) to better understand the attentional distribution to representations of comprehension and speech planning stimuli, and how they interact in the neural signal. To do this, we leveraged the picture-word interference (PWI) paradigm with delayed naming, where participants simultaneously comprehend auditory distractors (auditory [f1]; tagged at 54 Hz) while preparing to name related or unrelated target pictures (visual [f2]; tagged at 68 Hz). RIFT elicits steady-state evoked potentials, which reflect allocation of attention to the tagged stimuli. When representations of the tagged stimuli interact, increased power has been observed at the intermodulation frequency resulting from an interaction of the base frequencies (f2 ± f1; Drijvers et al., 2021). Our results showed clear power increases at 54 Hz and 68 Hz during the tagging window, but no power difference between the related and unrelated condition. Interestingly, we observed a larger power difference in the intermodulation frequency (compared to baseline) in the unrelated compared to the related condition (68 Hz − 54 Hz: 14 Hz), indicating stronger interaction between unrelated auditory and visual representations. Our results go beyond standard PWI results by showing that participants’ difficulties in the related condition do not arise from allocating attention to the pictures or distractors. Instead, processing difficulties arise during interaction of the concepts or lemmas invoked by the two stimuli, thus, we conclude, that interaction might be downregulated in the related condition.

## INTRODUCTION

A robust finding of picture-word interference (PWI) studies is the semantic interference effect: Speakers are slower to respond when naming target pictures that are presented with categorically related than with unrelated auditory or written distractors (for review see Bürki et al., 2020). The PWI literature usually focuses on identifying the timing of the interference effect, arising from competition between related words during lexical selection (e.g., Schriefers et al., 1990) or from the need to remove related distractors from a response buffer (e.g., Finkbeiner & Caramazza, 2006). The semantic interference effect usually disappears with delayed naming of the pictures, as the competition between the picture name and distractor is resolved within the delay window (e.g., Mädebach et al., 2011; Piai et al., 2011). Given that immediately after stimulus presentation the representations of target and distractor compete on some level, the presence of the target influences the neural representation and the processing of the distractor and vice versa, meaning that the representations interact. In this study, we leverage the PWI paradigm to better understand the attentional distribution between comprehension and speech planning, and their interaction, as in this paradigm participants simultaneously comprehend auditory distractors while preparing to name picture targets. We investigated how participants distributed their attention to representations of speech planning and comprehension stimuli. Crucially, we also investigated how these representations interacted in the neural signal.

Speech planning as well as listening requires attentional capacity (Cook & Meyer, 2008; Ferreira & Pashler, 2002; Jongman et al., 2015; Kristensen et al., 2013; Moisala et al., 2015), which means that attentional resources are shared between speech planning and comprehension when they take place concurrently. In line with this, evidence from dual-tasking studies shows that there is mutual interference between speech planning and comprehension (Bögels et al., 2015; Daliri & Max, 2016; Fargier & Laganaro, 2016, 2019). Does the content of speech planning and comprehension affect the attentional distribution toward these processes? In many dialogues, the planned utterance depends on the content of comprehension. This means that interlocutors use the semantics of comprehended speech to inform the semantics of speech planning (Bögels et al., 2015, 2018). Thus, relatedness of the speech planning and comprehension streams might affect how speakers distribute their attentional resources toward these processes.

There is some evidence that the comprehension and production systems work in unison. Several theories assume that the two systems work jointly to improve their functioning (Chang, 2002; Federmeier, 2007; Pickering & Gambi, 2018; Rohde, 2002). For example, the production system can aid with predicting upcoming words during comprehension (Gastaldon et al., 2020, 2023; for review see Gastaldon et al., 2024). Further evidence for the connection between the systems comes from PWI and priming studies, which show that the representations for speech planning and comprehension interact, as comprehending semantically related distractors or primes influences the speed of picture naming (for reviews see Bürki et al., 2020; Lucas, 2000). However, it is still debated when this interaction arises (Abdel Rahman & Melinger, 2019; Dell’Acqua et al., 2007; Piai et al., 2011; Roelofs, 1992; van Maanen et al., 2009), and which further processes (e.g., perceptual attention, other stages of speech planning) could influence this interaction. Additionally, the nature of the interaction between the systems may depend on the experimental task or the context in which the systems interact. Thus, the potential interaction between the representations within the comprehension and production system is not fully understood. To a large extent, this is because it is difficult to study the interaction between covert complex processes.

A novel approach, rapid invisible frequency tagging (RIFT), has recently been put forward for investigating both covert attentional allocation to multiple stimuli and stimulus interaction (Brickwedde et al., 2025; Duecker et al., 2021; Ferrante et al., 2023; Pan et al., 2021; Seijdel et al., 2023, 2024; Zhigalov et al., 2019, 2021; Zhigalov & Jensen, 2020). In this method the luminance of the visual stimuli and the amplitude of the auditory stimuli are periodically modulated at high frequencies (>50 Hz), which leads to imperceptible tagging (imperceptibility of auditory tagging was tested in unpublished pilots: Drijvers et al., 2021; for imperceptibility of visual tagging see Minarik et al., 2023, and Spaak et al., 2024). Visual and auditory tagging produce robust steady-state evoked potentials (SSEP). The strength of these potentials reflects visual or auditory attention toward the tagged stimuli (Toffanin et al., 2009). Tagging two stimuli at different frequencies creates increased power at intermodulation frequencies, which results from the nonlinear interaction of the base frequencies (f2 ± f1; e.g., Regan et al., 1995). The power at the intermodulation frequency is thought to reflect the strength of interaction between the representations of the two tagged stimuli (Drijvers et al., 2021; Seijdel et al., 2024). Previous studies have used the intermodulation frequency to tap into audio-visual integration (Drijvers et al., 2021). In the visual domain, using a low-frequency tagging approach, the intermodulation frequency is thought to reflect high-level visual computations, such as perceptual learning (Vergeer et al., 2018) or perceptual binding (e.g., Alp et al., 2016; Boremanse et al., 2014; Formica et al., 2024). For example, Formica and colleagues found that the similarity of hand movements in videos tagged at different frequencies affected the strength of the intermodulation peak. In the present study, we utilized the RIFT approach to study the interaction between representations evoked by the perceptual features of the target picture and distractor word stimuli in the picture-word interference paradigm. Here, the target picture and distractor word stimuli served as proxies for speech planning and comprehension, while the tagging frequencies, as well as the intermodulation frequency, served as markers that could be used to study the attention allocation to and interaction of the stimuli.

To study speech planning during comprehension, we leveraged a delayed-naming PWI paradigm combined with a novel combination of RIFT and electroencephalography (EEF). In previous work, RIFT has been used to study cognitive phenomena with magnetoencephalography (MEG), but not with EEG (but see for the feasibility of combining RIFT and EEG Arora et al., 2024). This study therefore serves as a proof-of-principle of combining RIFT with EEG in more complex experimental paradigms. In this study, participants named visually tagged pictures (68 Hz luminance modulation) that were presented simultaneously with auditorily tagged distractor words (54 Hz amplitude modulation). We examined the SSEPs at the tagging frequencies to determine whether relatedness affected how participants distributed their attentional resources to speech planning stimuli (i.e., target picture) and to comprehension stimuli (i.e., distractors). More attention toward a stimulus would be reflected in higher SSEPs. Importantly, this study also examined the intermodulation frequency to explore the interaction between the target pictures and the auditory distractors. Thus, with the help of the intermodulation frequency we examined the interaction between speech planning and comprehension representations to determine whether the speech planning and comprehension representations interacted differently depending on their relatedness.

## MATERIALS AND METHODS

Data and analysis scripts are available at https://osf.io/nzp6u/. The present study was approved by the Ethics Committee of the Social Sciences department of the Radboud University Nijmegen (ECSW-2019-019).

### Participants

Thirty-one right-handed, native Dutch-speaking participants without hearing or language impairments and normal or corrected to normal vision took part in the experiment for financial compensation. Data from one participant were excluded because there were too many muscle artifacts. The remaining 30 participants had a mean age of 23.13 years (range: 18–32) and 11 were male.

### Materials

For the pretest, 52 categorically related picture distractor pairings were taken from previous studies (Mahon et al., 2007; Schriefers et al., 1990) or were newly created (see Supplementary Materials, available at https://doi.org/10.1162/nol_a_00171). The present study needed longer distractors than standard PWI studies to ensure the speech input was long enough for reliable auditory tagging and the subsequent analysis. To ensure that a sufficient number of iterations of the tagging would be included during the presentation of the auditory stimulus, we only used auditory distractors that were longer than 700 ms. All distractors were recorded in Audacity 3.0.0 by a female speaker who spoke slowly. The log 10-word frequencies (WF) for the distractors and picture names were calculated using SUBTLEX-NL (Keuleers et al., 2010). The mean WF of the distractors was 2.09 (range: 0.48–3.59); for the picture names it was 2.79 (range: 1.15–5.11). The distractors were paired with different pictures from our set to create the unrelated picture distractor pairs. This was done to find pairings with related and unrelated distractors of similar length and WF per picture. Thus, each picture was paired with one categorically related and one unrelated distractor. The pictures were taken from the BOSS photograph database (Brodeur et al., 2014) as well as open access online sources.

The PWI materials were tested in a behavioral pretest with 14 different native Dutch participants. In the pretest participants were familiarized with the picture names and the auditory distractors. They read through all the picture names, which were printed underneath the pictures, and they listened to a recording of all of the distractors in a random order. In the behavioral pretest, each target-distractor pair was presented twice. Participants first saw a fixation cross for 700 ms, after which the target picture appeared simultaneously with the auditory distractor. The participants’ task was to name each picture as fast as possible.

Trained research assistants transcribed the responses and determined the reaction times or naming latencies in Praat (Boersma & Weenink, 2023). They were blind to the experimental conditions and questions. We excluded incorrect responses and responses with naming latencies >2.5 *SD* from the mean per condition. Naming latencies were analyzed with a generalized linear mixed-effects model (GLMM) fitted with the lme4 package (Version 1.1.31; Bates et al., 2015). The model included condition as a fixed factor and random intercepts and slopes for condition by participant (1 + condition | participant), as models with more complex random structures had convergence issues. This model utilized Gamma family with an identity link to account for the strictly positive and right-skewed distribution of naming latencies. We increased the maximum number of iterations for the optimizer (bobyqa) to 10^5^ as initial attempts to fit the model using the default optimizer failed to converge due to model complexity. The average naming latency was shorter in the unrelated condition (*M* = 721.47 ms, *SD* = 165.44 ms) than in the related condition (*M* = 738.70 ms, *SD* = 181.94 ms, *β* = −18.81, *SE* = 5.26, *t* = −3.57, *p* < 0.001). This result replicates the well-established semantic interference effect in the PWI paradigm (see Bürki et al., 2020), which in current models of lexical access is attributed to stronger competition between related than between unrelated distractor-target pairs.

Based on the pretest, we selected 32 targets that showed the strongest interference for the EEG experiment. The average naming latencies for this subset were 718.76 ms (*SD* = 161.47 ms) in the unrelated condition and 739.74 ms (*SD* = 181.96 ms) in the related condition. The mean duration of the distractors was 876.88 ms (range 722–1,044 ms). Each target-distractor pair was presented twice during the EEG experiment, with at least 10 intervening trials. We created 30 pseudorandomized lists with MIX (van Casteren & Davis, 2006). The same condition occurred maximally on four consecutive trials.

### EEG Acquisition

EEG was recorded using 32 electrodes (actiCap, Brain Products, Germany) arranged according to the 10–20 system. Thirty-one electrodes were positioned on the scalp and one electrode was placed on the left mastoid and later used for rereferencing. The right mastoid was used as an online reference. The data was recorded using a 1000 Hz sampling rate. We adjusted the impedances of all electrodes below 10 kΩ, and the ground electrode was placed in the AFz position.

### Procedure

The participants were first asked to familiarize themselves with the picture names by looking at each picture and reading its associated name on a printed sheet. All of the experimental stimuli were presented using MATLAB 2023b (Mathworks, 2023) and the Psychophysics Toolbox (Version 3.0.17; Brainard, 1997; Kleiner et al., 2007). Each picture was presented at a visual angle of approximately 7.13° (height) × 8.39° (width) at a viewing distance of approximately 80 cm. The stimuli were projected with a PROPixx DLP LED projector (VPixx Technologies Inc., Saint-Bruno-de-Montarville, Canada) and using a GeForce GTX960 2GB graphics card with a refresh rate of 120 Hz. This setup could achieve a presentation rate up to 1440 Hz, as the projector interprets the four quadrants and three-color channels of the GPU screen buffer as frames, that can be projected in rapid succession (4 quadrants * 3 color channels * 120 Hz = 1440 Hz).

**Figure 1.**
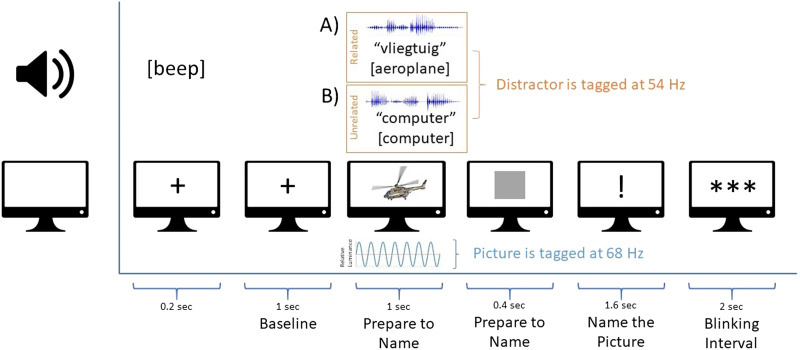
Experimental paradigm. Participants saw pictures with luminance modulations of 68 Hz while listening to auditory distractors that included amplitude modulations at 54 Hz. The auditory distractors were either (A) categorically related or (B) unrelated to the target pictures. Participants were asked to prepare the picture names as soon as possible, but only to say them out loud at the onset of the exclamation mark.

The experiment took place in a dimly lit room. During the task, participants first saw a fixation cross presented together with a beep for 200 ms, which was followed by the baseline period of 1,000 ms, during which participants only saw a fixation cross. Then participants saw a picture for 1,000 ms (i.e., tagging window). The luminance of the pixels of this picture were multiplied with a 68 Hz sinusoid (i.e., visual frequency tagging), phase-locked across trials. Participants were instructed to prepare the picture names as soon as possible in order to speed up naming after the response signal. The auditory distractors were presented simultaneously with the pictures. The amplitude of the auditory distractors was multiplied with a 54 Hz sinusoid (i.e., auditory frequency tagging). Subsequently, participants saw a gray square for 400 ms, after which an exclamation mark appeared for 1,600 ms. Participants were instructed to name the picture as fast as possible after the onset of the exclamation mark. After that, participants could blink at the onset of the three stars (see Figure 1).

### EEG Preprocessing

We analyzed all EEG data using FieldTrip (Version 20210325; Oostenveld et al., 2011) in MATLAB 2020b (Mathworks, 2020). The data was segmented from 1,000 ms before to 1,000 ms after target picture onset. Channels with excessive noise were removed (no more than four channels were excluded per participant). All channels were re-referenced to an average mastoid reference. We filtered the data with a band-pass filter between 0.1 and 100 Hz, and a notch filter at 50 Hz, 100 Hz, and 150 Hz to remove the line noise and its harmonics. We visually inspected the data and removed the segments containing non-physiological artifacts, resulting from other devices or electrode pops. Independent component analysis (ICA) using the runica algorithm was performed to correct for electrooculography artifacts and steady muscle activity. The remaining physiological artifacts were removed manually. The individual removed EEG channels were interpolated by a weighted average of the data from neighboring channels of the same participant. After the preprocessing, there were on average 54.93 trials (*SD* = 3.76) per participant in the related condition and 55.52 trials (*SD* = 3.50) in the unrelated condition.

### Analysis

The behavioral responses were scored as correct if participants named the picture with the familiarized name or its synonym. Omissions, names of other targets, or early responses were excluded from all analyses (1.07% of the trials). The reaction times or naming latencies from the EEG experiment were determined based on an automatic threshold, as we did not expect any differences between conditions in a delayed naming experiment. Trials where the automatic threshold failed to detect a response were excluded from the behavioral analyses (3.04% of the trials in related condition, and 3.06% in the unrelated condition). Additionally, we excluded responses with naming latencies >2.5 *SD* from the mean per condition for the behavioral analysis. Naming latencies were analyzed with a GLMM fitted with the lme4 package. The model included condition as a fixed factor and random intercepts and slopes for condition by participant (1 + condition | participant). For optimal comparability of the results, we utilized the same model parameters as for the pilot analysis. To examine the success of the frequency tagging at the base frequencies we performed both power and coherence analysis. The coherence analysis did not show different results compared to the power analysis; thus, we only describe it in the Supplementary Materials. The power spectra were calculated per participant and per condition between 1 and 80 Hz using 1 Hz steps for the tagging interval as well as the baseline. Each frequency step was modulated with a boxcar taper, followed by the Fourier transform of the tapered signal. We computed averages per condition for every participant.

We first evaluated power differences between the tagging window and the baseline at the main tagging frequencies (i.e., 68 Hz and 54 Hz) and at the intermodulation frequency (i.e., 14 Hz) using two-tailed nonparametric cluster-based permutation tests (Maris & Oostenveld, 2007). We expected to only find the low intermodulation peak at 14 Hz as responses in higher frequencies (i.e., at the sum of the tagged signals, 122 Hz) tend to be weaker and difficult to detect with EEG. Following previous studies, we also expected to only find the intermodulation peak in the power analysis (Drijvers et al., 2021; Seijdel et al., 2024), as the intermodulation frequency might have weaker phase coherence across trials than the main tagging peaks, especially if the moment of interaction between the stimuli may vary. This test computed dependent samples *t* tests between the conditions for the frequency of interest and for all electrodes. If two or more neighboring electrodes reached significance, they formed a cluster. Subsequently, a sum of all the *t* values of each cluster was calculated. To control for the family-wise error rate, the two conditions were combined and subsequently randomly separated into two artificial groups 5,000 times. The summed *t* values of all of the randomly generated clusters were computed in the same way as described above. Subsequently, a distribution of these summed *t* values was generated. Comparing the summed *t* values computed based on the original clusters to this distribution resulted in Monte Carlo significance probabilities, which were considered significant if smaller than 0.05. The differences between related and unrelated conditions were examined only after the tagging peaks significantly differed from the baseline.

To compare power differences between the conditions, we first computed the percentage power change between the tagging window and the baseline. The percentage power change was calculated as ((Pow during tagging window − Pow during baseline) / Pow during baseline) * 100. The condition differences were also tested with nonparametric cluster-based permutation tests using the same procedure described above.

## RESULTS

### Behavioral Analysis

Less than 2% of responses were marked as incorrect and excluded from all further analyses. In line with previous studies that used the PWI paradigm with delayed naming (Mädebach et al., 2011; Piai et al., 2011), we found that the naming latencies did not significantly differ in the related (*M* = 505.31 ms, *SD* = 164.25 ms) as compared to the unrelated condition (*M* = 513.38 ms, *SD* = 162.31 ms, *β* = 5.30, *SE* = 5.28, *t* = 1.00, *p* = 0.315). Note that we found a significant interference effect in our pretest, where participants used immediate naming. This indicates that the absence of a significant interference effect does not stem from insufficient sensitivity of the experimental materials. Instead the interference effect was absent because the competition between target and distractor had been resolved during the delay period and the target had been selected. Thus, our results indicate that participants, as requested, started speech planning before the response period.

### Main Frequency-Tagging Peaks

**Figure 2.**
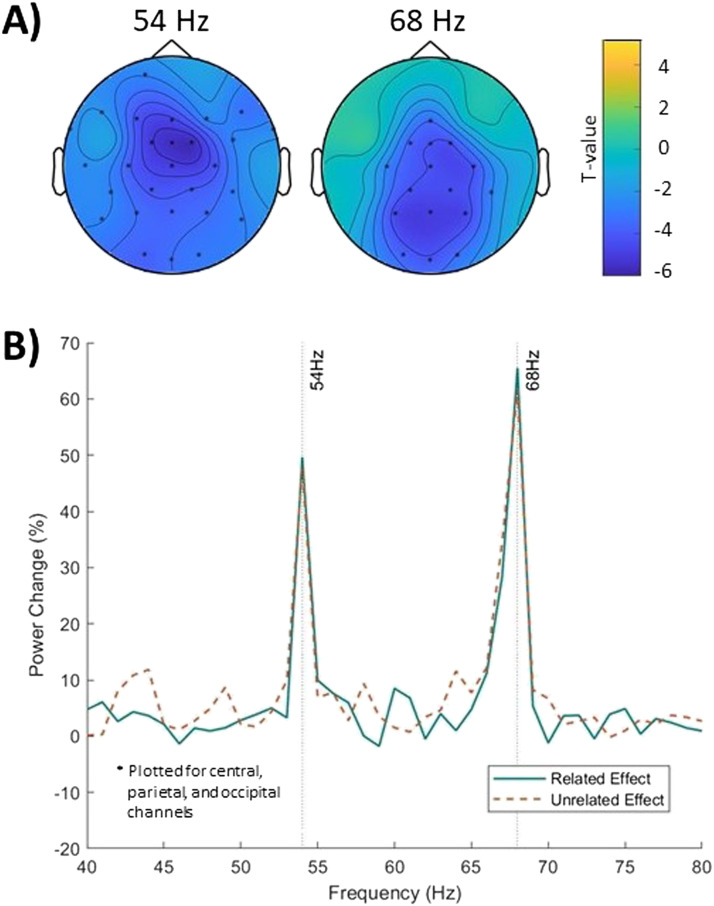
Power analysis. (A) Shows that the percentage power change between tagging window and baseline was significant at 54 Hz and 68 Hz. The electrodes for which the contrast was significant are highlighted with asterisks. (B) Shows the percentage power change plotted separately for related (green solid) and unrelated (orange dashed) condition, plotted for average of central, parietal, and occipital electrodes. The percentage power change did not differ between related and unrelated conditions at 54 Hz or 68 Hz.

We first examined whether the visual and auditory SSEP were larger in the tagging window compared to the baseline. The nonparametric cluster-based permutation test (for both conditions combined) showed that the visual SSEP arising from the tagging at 68 Hz resulted in a significant power peak in the tagging window compared to the baseline (*p* < 0.001). Based on visual inspection about 63% of the participants showed the visual tagging peak, similar to what has been reported for MEG data in Pan et al. (2021). The auditory SSEP arising from the tagging at 54 Hz also showed significant power peak in the tagging window compared to the baseline (*p* < 0.001). Based on visual inspection about 77% of the participants showed the auditory tagging peak. The visual SSEPs were significantly different from baseline mainly in the occipital and central electrodes, while the auditory SSEPs were significantly different from baseline in most electrodes (see Figure 2A).

Subsequently, we computed the percentage power change between the tagging window and the baseline for both conditions (Figure 2B). The nonparametric cluster-based permutation tests found no clusters between the conditions, this shows that neither the visual SSEP or the auditory SSEP differed between the related and unrelated condition.

### Intermodulation Frequency

For the intermodulation frequency at 14 Hz, we also examined whether the SSEPs were larger in the tagging window compared to the baseline. The nonparametric cluster-based permutation test showed significant clusters in frontal and central electrodes between the tagging window and the baseline (*p* < 0.001).

To determine which electrodes should be analyzed to examine the differences between the conditions at the intermodulation peak, we followed a similar approach as Ferrante et al. (2023). Per participant, we selected six electrodes that showed the largest percentage power difference between the baseline and tagging window at 14 Hz. Based on the neighborhood structure, we determined which of the six electrodes neighbored one another. We chose the largest cluster from these electrodes for further analysis. We also performed two validations of this electrode selection approach (see Supplementary Materials for detailed description and results). (1) We performed the electrode selection at the main tagging frequencies. The results showed that at 68 Hz and 54 Hz occipital and central electrodes were selected respectively as the regions with the largest effects, supporting our main results. (2) We performed the same selection procedure at the neighboring frequencies of the intermodulation frequency (i.e., 12 Hz, 13 Hz, 15 Hz, and 16 Hz). The power peak was most pronounced at 14 Hz compared to the peaks at the neighboring frequencies, confirming a clear intermodulation peak at 14 Hz.

**Figure 3.**
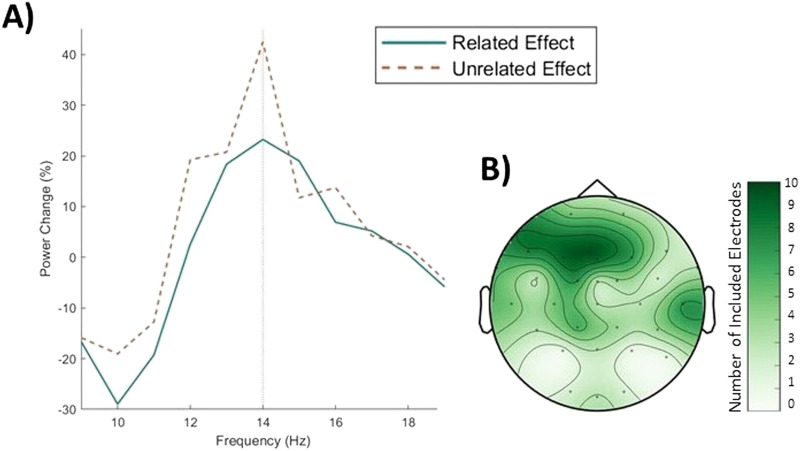
Power analysis for electrodes that show largest effect at 14 Hz. (A) Shows the percentage power change plotted separately for related (green solid) and unrelated (orange dashed) condition. (B) Shows the electrodes for which the power effect is plotted. The electrodes were calculated based on the largest percentage power change between tagging window and baseline (per participant) calculated for 14 Hz for both conditions combined. The color scale shows the number of electrodes included at each location.

Figure 3B shows the electrodes that were selected for the analysis at 14 Hz. Of the channels selected for the 14 Hz analysis, most were the left frontal electrodes. Subsequently, we averaged the signal over the selected electrodes based on the 14 Hz effect separately for the time periods of interest (i.e., baseline and tagging window) and the conditions (related and unrelated). We then computed a paired *t* test between related and unrelated condition for the percentage power difference. This analysis showed two important findings. (1) There was a clear peak precisely at 14 Hz for both related and unrelated conditions (Figure 3A), which was not present at neighboring frequencies (e.g., 13/15 Hz). This demonstrates that this peak reflects the intermodulation frequency, and not a more global oscillatory effect, which would span a wider range of frequencies (e.g., beta power differences resulting from a difference in the baseline and activity in the tagging window). (2) The *t* test showed higher steady-state responses at 14 Hz for the percentage power change in the unrelated compared to the related condition (*t*(29) = −3.02, *p* = 0.005, *d* = 0.55), which indicates that the representations of the visual and auditory stimuli interacted differently depending on their relatedness.

## DISCUSSION

In this EEG study, we utilized the RIFT method to ask (1) how participants distributed their attentional resources when they are planning to name a picture while hearing a related or unrelated distractor word and (2) how their representations of the target and distractor stimuli interacted on a neural level, based on relatedness. Our results showed that the power of the frequency-tagged signals did not significantly differ between the related and unrelated condition at either 68 Hz (i.e., visual tagging frequency) or 54 Hz (i.e., auditory tagging frequency). This indicates that relatedness did not affect attention to early stages of comprehension or speech planning stimuli. Crucially we found higher power at the intermodulation frequency at 14 Hz in the unrelated compared to the related condition, suggesting that there was stronger interaction between the representations of the target picture and distractor auditory word in the unrelated compared to the related condition.

### Relatedness Did Not Affect Early Attention to the Target Picture or Distractor Word

We looked at visual and auditory SSEPs to tap into visual attention to the speech planning stimulus and auditory attention to the comprehension stimulus, as the power of SSEPs is thought to relate to the amount of attention toward the tagged stimuli (Toffanin et al., 2009). We showed that neither the visual nor the auditory SSEPs significantly differed between the related and unrelated conditions. This indicates that the relatedness of the stimuli did not affect early visual and auditory attention. Similarly, in a previous RIFT study relatedness between words and gestures did not affect visual or auditory attention (Drijvers et al., 2021).

Our results are in line with the PWI literature. Classic accounts of PWI effects (e.g., Levelt et al., 1999; Roelofs, 1992; Schriefers et al., 1990) assume that the earliest parts of the picture naming process are independent of the relationship between target and distractor. These accounts posit that the interference effect arises in post-perceptual stages, either at the level of lexical selection (e.g., Levelt et al., 1999; Roelofs, 1992; Schriefers et al., 1990), which begins around 150 ms after picture onset (Indefrey & Levelt, 2004), or later at the level of response selection (e.g., Finkbeiner & Caramazza, 2006; Mahon et al., 2007), which takes place about 350 ms after picture onset (Indefrey & Levelt, 2004). In line with these accounts, participants in our experiment did not direct different amounts of auditory attention to the distractor, or visual attention to the target, depending on their relatedness.

### Interaction Between Visual and Auditory Representations Was Affected by Their Relatedness

This study also examined the intermodulation frequency that is thought to reflect the strength of the interaction between representations of the tagged stimuli (Drijvers et al., 2021; Seijdel et al., 2024). We examined whether and how the interaction between the speech planning and comprehension representations was affected by their relatedness. We tested whether the interaction was stronger in the categorically related condition, where the concepts invoked by target and distractor were similar, or whether the interaction was stronger in the unrelated condition, which was the easier condition, associated with faster naming latencies due to weaker competition between target and distractor (Bürki et al., 2020).

The central finding of this study is that the strength of the intermodulation peak did depend on the relatedness of the auditory and visual stimuli presented together. The intermodulation peak was higher in the unrelated compared to the related condition, suggesting that there was stronger interaction between representations of the visual stimulus guiding speech planning and the auditory stimulus in the unrelated condition. This is the first evidence indicating that semantic relatedness between the tagged stimuli affects the strength of the intermodulation frequency in clear speech.

At first glance, it might seem surprising that the interaction between the representations would be stronger in the unrelated condition, as the interaction is thought to be stronger when stimuli are more similar (however, see Formica et al., 2024, for similar unexpected results from the perceptual binding domain). However, this pattern of results becomes more intuitive when considered within the context of the PWI paradigm. Evidence from numerous studies including our own pre-test, has shown that picture naming is slower in the semantically related than in the unrelated distractor condition (Bürki et al., 2020). Most current accounts of this pattern agree that this semantic interference effect arises because there is competition between the distractor and target representations, which needs to be resolved before the target can be selected. This is because of mutual activation, causing the related target and distractor pairs to compete more strongly than unrelated ones (e.g., Abdel Rahman & Aristei, 2010; Canini et al., 2016; Oppenheim et al., 2010; Roelofs, 2018). Considering that our main experiment used the same materials as the pilot, it can be assumed that participants still experienced the conflict between the representations of the target and distractor during the tagging window and resolved it before the onset of the response period (e.g., Piai et al., 2011). It has been proposed that competition between representations is subject to top-down regulation or attentional control of the speech planning process, stemming from activation in the anterior cingulate cortex (ACC; Botvinick et al., 2001). In line with this proposal (called the conflict monitoring hypothesis), several PWI studies have found higher activation in the ACC in conditions with more competition (i.e., the related condition; de Zubicaray et al., 2001; Piai et al., 2013). In our study, this top-down regulation might have resulted in automatic suppression of the interaction between speech planning and comprehension representations in the related condition, and thus a lower intermodulation frequency.

While the current study suggests that semantic manipulations influence the intermodulation frequency, with higher values observed in the unrelated condition, contrasting findings in previous RIFT studies warrant further discussion. Notably, Drijvers et al. (2021) and Seijdel et al. (2024) found different results regarding how semantics, or more specifically, gesture-to-speech congruence, affected the strength of the intermodulation frequency (Drijvers et al., 2021; Seijdel et al., 2024). Specifically, Drijvers and colleagues, compared semantically congruent and incongruent speech gesture pairs (e.g., driving gesture paired with the verb “drive” versus the verb “eat”) and did not find a congruency effect on the intermodulation frequency. By contrast, in a similar study using degraded speech, Seijdel et al. (2024) found a higher intermodulation frequency when participants were presented with matching pairs of gestures and speech compared to mismatching pairs. This result seemingly contrasts with the present finding that the intermodulation frequency was higher in the unrelated condition than the related condition. However, a direct comparison between the results of these studies is problematic because they differed in many potentially relevant ways. For instance, the current study used two types of mismatching stimulus pairs, semantically related and unrelated ones, whereas Seijdel and colleagues compared mismatching and matching pairs. Furthermore, in the present study the stimuli were presented in clear speech, whereas Seijdel and colleagues used degraded and clear speech, and found an effect of condition of the intermodulation frequency in the degraded speech condition. Finally, the current study used a novel task—preparing to name pictures while listening to words, which had a stronger focus on the visual stimulus, whereas Seijdel and colleagues studied the processing of speech and co-speech gestures, with a stronger focus on the auditory stimulus. Which of these differences, if any, affected the direction of effect of stimulus pairing on the intermodulation frequency, needs to be investigated in further work.

It is still debated which processes give rise to and affect the intermodulation peak. Previous studies took the intermodulation frequency to represent the strength of interaction between the representations of the two stimuli (Drijvers et al., 2021; Seijdel et al., 2024). Our results indicate that if the intermodulation peak indeed represents ease of interaction (i.e., higher power reflects stronger interaction), the intermodulation peak must be affected by top- down regulation or executive control processes that lead to suppressed interaction in the related condition. Future studies, using higher density of electrodes or MEG, could link the strength of the intermodulation peak to alpha power. This would be useful as an increase in alpha power is thought to be a marker of executive control processes or functional inhibition (e.g., Klimesch et al., 2007; Mazaheri et al., 2014; Zanto et al., 2011). High-density EEG/MEG studies could investigate whether higher alpha power over task-relevant brain regions reflects increased top-down regulation in the related condition, and whether this alpha power increase relates to the strength of the intermodulation frequency. The correlation between the intermodulation frequency and alpha power should be stronger in conditions that require more top-down regulation, such as the related, compared to the unrelated condition in PWI studies.

### Implications for Speech Planning and Comprehension

This study offers evidence that representations arising from comprehension and speech planning streams interact on a neural level. This points to the conclusion that the comprehension and speech production streams are linked through semantic representations that interact between the streams. This is consistent with the widely held view that conceptual representation and lemmas (grammatical word representations) are shared between production and comprehension systems (e.g., Levelt et al., 1999; McQueen & Meyer, 2019). In this study, the interaction was present even though processing of the stimuli in the comprehension stream was not task-relevant, which speaks to the automaticity of this interaction.

Behavioral evidence from PWI and priming studies has shown that the representations of speech planning and comprehension interact (for reviews see: Bürki et al., 2020; Lucas, 2000), but this interaction is difficult to examine with more naturalistic comprehension and speech planning materials. In dialogue, interlocutors’ utterances usually consist of several words or even sentences that are often planned in response to a comprehension stream also consisting of multi-word or multi-sentence utterances. Thus, it remains unclear which speech planning representations and which comprehension representations interact and when. Our results showed that RIFT can be a useful tool to study the naturalistic interactions between speech planning and comprehension representations, as almost any section of the comprehension stream and visual input could be tagged, and subsequently tracked in the neural signal. Thus, further RIFT studies could investigate how the interaction between speech planning and comprehension is carried out in more naturalistic dialogue settings.

The results of the current study suggest several directions for further research. For example, one may ask whether speech planning is crucial for the occurrence of the integration process between the auditory and visual representations, or whether one would see the same interaction when participants only passively view the pictures or categorize them (e.g., as natural versus man-made) using manual responses. It is therefore challenging to disentangle whether the observed effects reflect attention allocation and interaction processes during early perceptual and conceptual stages of processing the target picture, or whether they specifically index later stages of speech planning. The picture itself, while the target for the production stream, is also part of the comprehension stream, as recognizing the picture and accessing the conceptual representation are necessary precursors to naming. Future studies using a context-induced picture naming paradigm, where the predictability of the picture can modulate the extent of speech planning before picture onset, may help distinguish these possibilities. Furthermore, the present study examined the interaction between speech planning and comprehension when both stimuli were nouns. However, in PWI studies, interference effects are influenced by multiple factors beyond the semantic relationship between the target and distractor. One key factor is the grammatical category (parts of speech) of the target and distractor (Fang et al., 2015; Mahon et al., 2007). Future RIFT studies could present participants with object pictures (e.g., “bed”) and distractor nouns (e.g., “chair”) and verbs (e.g., “sleep”). This could shed light on how grammatical category affects the interactive processes. In PWI studies, using related verb distractors sometimes yields facilitation rather than interference (Fang et al., 2015; Mahon et al., 2007). Thus, using object pictures with noun and verb distractors in a RIFT study could help establish whether the strength of the intermodulation peak tracks the naming latencies. If this is the case, we should see a higher intermodulation peak for the related than unrelated verb distractors.

### Implications for Future RIFT Studies

The present study is the first study that combined the RIFT method with EEG (instead of MEG) to study cognitive phenomena in a complex experimental paradigm (but see Arora et al., 2024, for an elegant visual perception study). Our results show the robustness of the SSEPs as well as the intermodulation frequency across these neuroimaging tools. In the current study, the intermodulation peak was set at a higher frequency (i.e., 14 Hz) than in the earlier studies, which used frequencies of 7 Hz and below (Drijvers et al., 2021; Seijdel et al., 2024). Together, these findings show the flexibility as well as reliability of the RIFT methodology. While in the present study we focused on the intermodulation frequency at f2–f1, future studies could potentially examine a broader range of intermodulation frequencies to more comprehensively assess the nonlinear interactions between the representations invoked by the target and distractor stimuli. Here, it is important to note that when using higher frequencies as the base frequencies for tagging, the higher-order intermodulation frequencies (e.g., 2f1–f2) could be difficult to detect, as they often result in frequencies where RIFT induced brain responses have been found to be weak (see for recommendations: Minarik et al., 2023).

Importantly, this is the first study to show that semantic relatedness between the tagged stimuli alone affects the intermodulation frequency. Other studies have shown that the strength of the intermodulation peak was sensitive to the ease of lower-order audiovisual information processing (Drijvers et al., 2021; Seijdel et al., 2024). Our results indicate that the intermodulation peak represents more than a low-level interaction between the visual and auditory representations. Instead the intermodulation peak is sensitive to an interaction of the representations invoked by the stimuli on a semantic level as well.

### Conclusion

Our results are in line with previous behavioral work demonstrating that related distractors hinder picture naming relative to unrelated ones (Bürki et al., 2020). Our results go beyond this work by showing that participants do not have more difficulty visually attending to the related pictures or inhibiting the related auditory distractors. This is in accordance with the classical PWI accounts (e.g., Levelt et al., 1999; Roelofs, 1992; Schriefers et al., 1990), predicting that the interaction between the targets and distractors arises at the lexical level. Our results show that processing difficulties arise when the two representations of the stimuli interact. The competition between the representations might be subjected to top-down control (Botvinick et al., 2001), especially in the related condition (de Zubicaray et al., 2001; Piai et al., 2013). This top-down regulation might have resulted in automatic suppression of the interaction between speech planning and comprehension representations in the related condition, and subsequently, a lower intermodulation peak. This study demonstrates that RIFT in combination with EEG is a suitable approach to investigate interactive processes between speech planning and comprehension. Importantly, this study offers evidence indicating that semantic relatedness between the tagged stimuli alone affects the intermodulation frequency.

## ACKNOWLEDGMENTS

We thank Caitlin Decuyper for assisting with stimulus creation; Sophie Slaats and members of the Communicative Brain group for helping during the piloting phase; student assistants from the Psychology of Language department for the help with pilot transcriptions; and all the members of the Psychology of Language department for valuable input on earlier versions of this project. The study was part of the first author’s PhD project, funded by the Max Planck Society, Munich, Germany.

## FUNDING INFORMATION

Cecília Hustá, Max Planck Instituut voor Psycholinguïstiek (https://dx.doi.org/10.13039/501100023341).

## AUTHOR CONTRIBUTIONS

**Cecília Hustá**: Conceptualization; Formal analysis; Project administration; Visualization; Writing – original draft; Writing – review & editing. **Antje Meyer**: Conceptualization; Writing – review & editing. **Linda Drijvers**: Conceptualization; Formal analysis; Writing – review & editing.

## DATA AND CODE AVAILABILITY

Data and analysis scripts are available on https://osf.io/nzp6u/.

## Supplementary Material



## TECHNICAL TERMS

Semantic interference effect: The finding that naming a target picture is slower when paired with a semantically related distractor compared to an unrelated one.
Lexical selection: The process of choosing the appropriate word from the mental lexicon during speech production.
Delayed naming: A task where participants wait for a specific cue before naming a target, allowing time to resolve competition between target and distractor representations.
Rapid invisible frequency tagging: A method where stimuli are modulated at high, imperceptible frequencies to track attention allocation toward the stimuli.
Steady-state evoked potentials: Brain responses that are elicited by stimuli presented at a constant frequency, used to measure attention allocation.
Intermodulation frequency: Frequencies that result from the nonlinear interaction of base tagging frequencies, used to measure the interaction between neural representations of different stimuli.
Luminance modulation: Periodic changes in the brightness of a visual stimulus, used in RIFT to tag and measure attention toward visual inputs.
Amplitude modulation: Periodic changes in the intensity of an auditory stimulus, used in RIFT to tag and measure attention toward auditory inputs.
