# Supplementary material for: Using Rapid Invisible Frequency Tagging (RIFT) to Probe the Neural Interaction Between Representations of Speech Planning and Comprehension
Source: Neurobiol Lang (Camb). 2025 Jul 15;6:nol_a_00171. doi: 10.1162/nol_a_00171 (PMC12307024; doi:10.1162/nol_a_00171)
Supplement: Supplementary file 1 [file nol-6-1-171-s001.pdf]

Using rapid invisible frequency tagging (RIFT) to probe the attentional distribution between speech planning and comprehension

Abbreviated Title: Neural interaction of speech planning and comprehension

1. Stimulus list

	Target	Target Translation	Target WF	Related Dist.	Related Dist. Translation	Related Dist. WF	Unrelated Dist.
1	dokter	doctor	4.0283	verpleegster	nurse	2.8351	tijdschrift
2	boek	book	3.8196	tijdschrift	magazine	2.6274	verpleegster
3	bezem	broom	2.2227	stofzuiger	hoover	2.0334	aardappel
4	wortel	carrot	2.4281	aardappel	potato	2.1673	stofzuiger
5	helikopter	helicopter	2.9814	vliegtuig	plane	3.5947	computer
6	tablet	tablet	2.0043	computer	computer	3.3212	vliegtuig
7	trampoline	trampoline	1.5315	glijbaan	slide	1.3617	croissant
8	stokbrood	baguette	1.3222	croissant	croissant	1.415	glijbaan
9	olifant	elephant	2.721	neushoorn	rhino	2.0414	ingenieur
10	wetenschapper	scientist	2.7275	ingenieur	engineer	2.415	neushoorn
11	meer	lake	4.9495	waterval	waterfall	2.248	plakband
12	ducttape	duct tape	na	plakband	band aid	2.0492	waterval
13	stropdas	tie	2.2945	sjaal	scarf	2.3674	nieren
14	longen	lungs	2.7324	nieren	kidneys	2.2577	sjaal
15	bed	bed	4.0209	zitbank	sofa	0.4771	telraam
16	rekenmachine	calculator	1.415	telraam	abacus	0.7782	zitbank
17	paard	horse	3.5632	kameel	camel	2.0755	autobus
18	trein	train	3.5051	autobus	bus	1.0414	kameel
19	handdoek	towel	2.6628	toiletpapier	toilet paper	1.9345	dobbelsteen
20	speelkaarten	cards	1.1461	dobbelsteen	dice	1.5051	toiletpapier
21	lamp	lamp	2.7839	schilderij	painting	2.9741	portemonnee
22	rugzak	backpack	2.5211	portemonnee	wallet	2.7404	schilderij
23	frisbee	frisbee	1.9085	springtouw	jump rope	0.8451	bodywarmer
24	jas	jacket	3.3233	bodywarmer	bodywarmer	na	springtouw
25	oor	ear	3.0394	tanden	teeth	3.2702	prinses
26	koning	king	3.7824	prinses	princess	3.269	tanden
27	kat	cat	3.364	papegaai	parrot	2.1614	nagels
28	haar	hair	5.1144	nagels	nails	2.7574	papegaai
29	ijsblokje	ice cube	1.2788	sneeuwbal	snow ball	1.6335	schildpad
30	krab	crab	2.2304	schildpad	turtle	2.2833	sneeuwbal
31	schep	shovel	2.3075	kruiwagen	wheelbarrow	1.7482	fotograaf
32	soldaat	soldier	3.3655	fotograaf	photographer	2.6375	kruiwagen

Table 1. Shows the stimuli used in the EEG experiment. The table includes the names of the picture targets, as well as the associated related and unrelated auditory distractors that were used. The table includes translations and word frequencies (WF) for the target and distractor names.

2. Coherence analysis

Coherence was calculated for every trial between the EEG signal and three dummy signals oscillating at 68 Hz, 54 Hz, and 14 Hz (Figure 1). The coherence measure was computed for frequencies between 0 and 80 Hz separately per baseline period and the tagging window using the boxcar filter and the mtmfft method. We evaluated coherence differences between the tagging window and the baseline at the main tagging frequencies (i.e. 68 Hz and 54 Hz) and at the intermodulation frequency (i.e. 14 Hz) using two-tailed non-parametric cluster-based permutation tests (Maris & Oostenveld, 2007) using 5000 permutations and the 0.05 significance threshold. The differences between related and unrelated condition were examined only after the tagging peaks significantly differed from the baseline.

Coherence at 68 Hz between the EEG signal and the 68 Hz dummy signal was significantly higher during the tagging window ($p < 0.001$). However, there were no clusters found when comparing the related and unrelated conditions. Similarly, coherence at 54 Hz between the EEG signal and the 54 Hz dummy signal was also significantly higher during the tagging window ($p < 0.001$), with no clusters found for the contrast between conditions. These results point to the same conclusions as the outcome of our main power analysis. Coherence at 14 Hz between the EEG signal and the 14 Hz sine wave was not significantly different between the baseline and the tagging window ($p = 0.09$). This is not surprising, as previous studies showed that the intermodulation peak does not manifest in coherence, but rather in power (Drijvers et al., 2021) especially when the moment of interaction might differ.

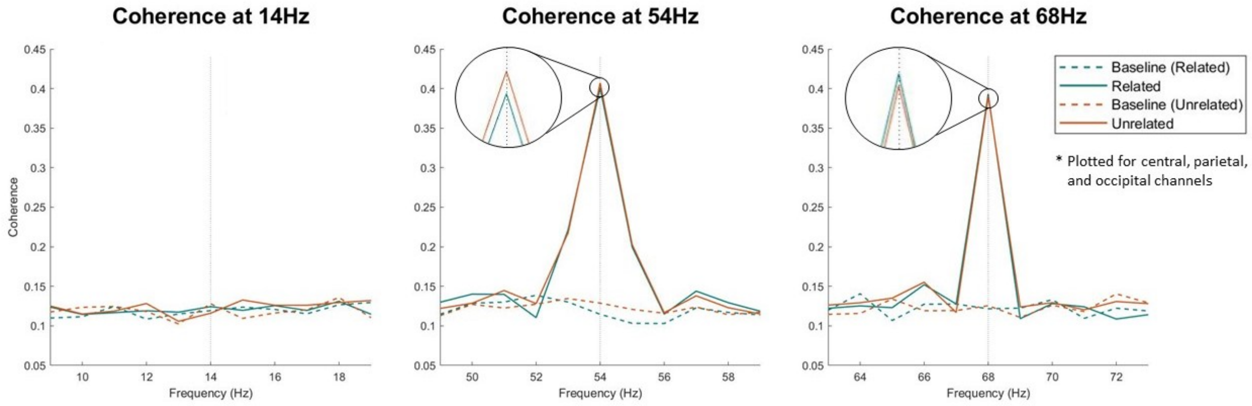

Figure 1. Coherence Analysis. *The plots show coherence between the EEG signal and 14 Hz (left plot), 54 Hz (middle plot), and 68 Hz (right plot) for the central, parietal and occipital channels. The plots include coherence during the baseline period (dashed lines) as well as during the tagging window (solid lines) for the related (green) as well as unrelated condition (orange).*

3. Validation of the electrode selection procedure

3.1. Selection of electrodes for the main tagging frequencies

As this is one of the first studies using RIFT with EEG, the standard analysis protocol has not yet been established. However, previous studies have analyzed RIFT data for different sensors per participant, depending on which sensor showed the strongest tagging response (Ferrante et al., 2023). Following Ferrante et al., 2023, we used a similar procedure to identify the intermodulation peak. Per participant, we selected 6 electrodes that showed the largest percentage power difference between the baseline and tagging window. From these electrodes, we chose the biggest cluster of electrodes based on the neighborhood structure for further analysis. For example, if all six electrodes were neighbors, or in other words electrodes in close proximity, all electrodes were included in the subsequent analysis, however, if one or two electrodes were located far away from the others, they would be excluded from the analysis. To establish whether this electrode selection procedure works, we have first performed it for the effects at 54 Hz and 68 Hz. Figure 2 shows that this analysis procedure selects mainly central electrodes for the 54 Hz effect and mainly occipital electrodes for the visual 68 Hz effect. Effects in these locations correspond well to where the auditory and visual effects should manifest in EEG. Thus, this electrode selection procedure could be used to capture the electrode cluster where the intermodulation peak manifests per participant.

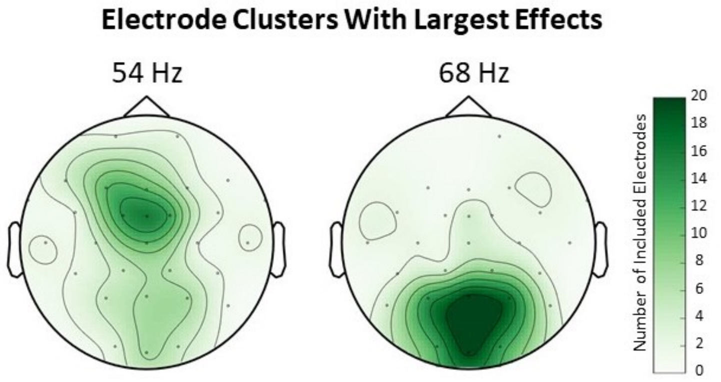

Figure 2. Electrodes That Show Largest Effect. *This plot shows the electrodes for which the power effect (((Pow during tagging window. – Pow during baseline) / Pow during baseline) *100) was the largest per participant at 54 Hz (left plot) and at 68 Hz (right plot). The color scale shows the number of electrodes included.*

3.2. Selection of electrodes at frequencies neighboring 14Hz

In the main manuscript we describe that the electrode selection procedure at 14 Hz results in a better-defined intermodulation peak. However, we wanted to establish how stable the intermodulation effect is, if the electrodes would be selected based on the biggest power change in neighboring frequencies. Thus, per participant, we computed electrode clusters that showed biggest power change at 12 Hz, 13 Hz, 15 Hz, and 16 Hz. We then plotted power change for each of these electrode selections. Figure 3 shows that the highest peak results at 14 Hz for the electrode cluster selected based on the largest difference between baseline and tagging window at 14 Hz. This supplementary analysis shows that employing a procedure to find electrodes where the intermodulation peak is most pronounced might be necessary when the intermodulation peak is investigated with EEG or in a paradigm associated with its own pattern of potentially overlapping oscillatory results.

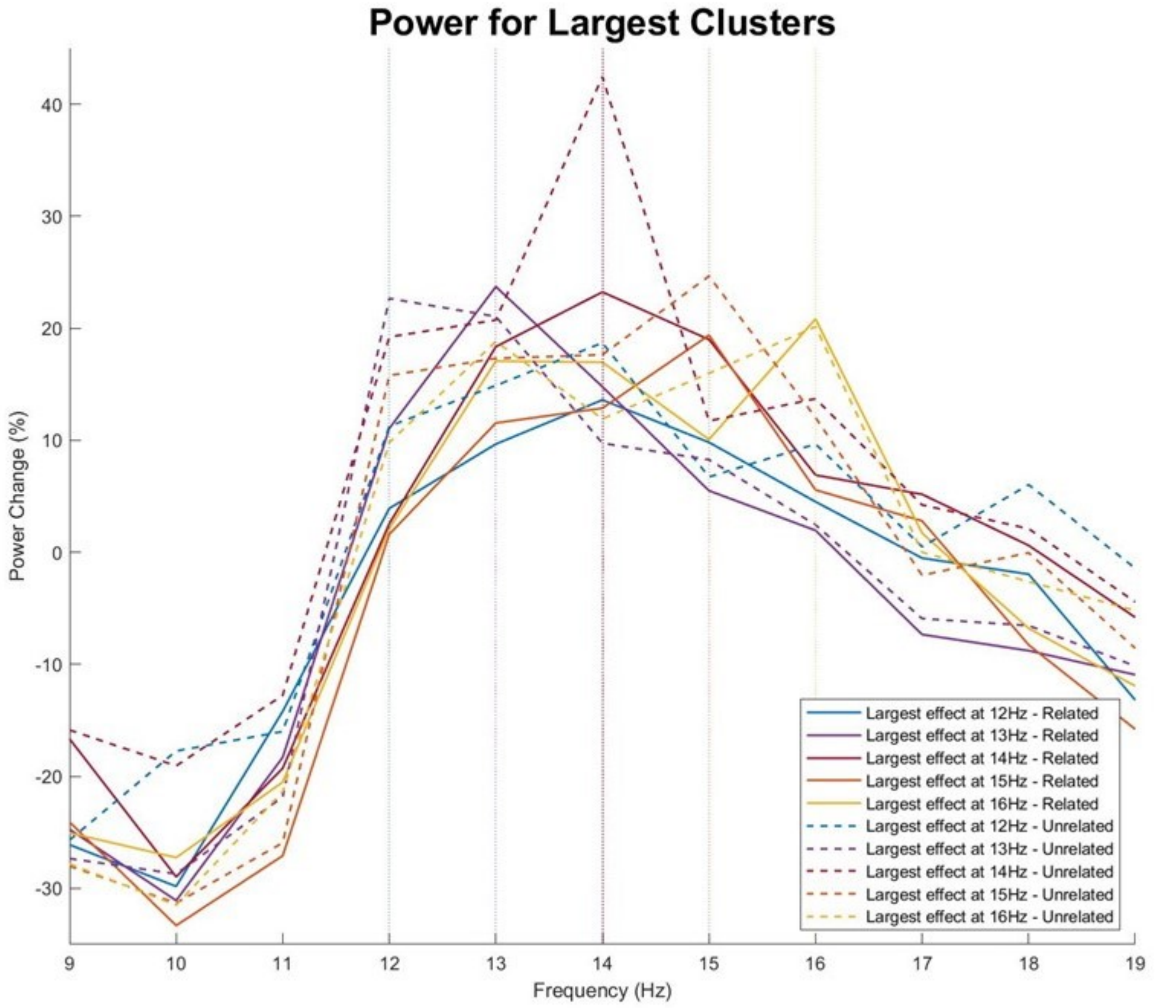

Figure 3. Power Analysis for Electrodes That Show Largest Effect at 12 Hz, 13 Hz, 14 Hz, 15 Hz, and 16 Hz. *This plot shows the percentage power change (((Pow during tagging window. – Pow during baseline) / Pow during baseline) *100) plotted separately for related (solid) and unrelated (dashed) condition. The percentage power change is plotted for clusters of electrodes that show largest effects between baseline and the tagging window at 12 Hz (blue), at 13 Hz (purple), at 14 Hz (red), at 15 Hz (orange) and at 16 Hz (yellow).*
